
JOURNAL INFORMATION
==============================
NLM Title Abbreviation: Behav Neurol
Journal ID: Behav Neurol
NLM Journal ID: 8914585
Journal ID: BN

Behavioural Neurology

ISSN: 0953-4180
EISSN: 1875-8584
Publisher: Wiley

ARTICLE INFORMATION
==============================
PMCID: PMC5434324
PMID: 21422568
DOI: 10.3233/BEN-2010-0308
Article ID: 343487
Article version: 1
Subjects: Research Article

Motion Influences Emotion, but Also Structural Facial Features Recognition

Sedda A. 1 *
Manfredi V. 1
Parente A. 1
Bottini G. 1 2
1 Psychology Department University of Pavia piazza Botta 6 27100 Pavia Italy
2 Cognitive Neuropsychology Center Niguarda Ca' Granda Hospital Milan Italy
*A. Sedda: sedda.anna@gmail.com
Print publication date: 2010
Electronic publication date: 2011 Mar 7
Volume: 23
Issue: 4
First page: 253
Last page: 254
Received 2011 Jan 31; Accepted 2011 Jan 31
Copyright: Copyright © 2010 Hindawi Publishing Corporation and the authors.
Copyright year: 2010
License: This is an open access article distributed under the Creative Commons Attribution License, which permits unrestricted use, distribution, and reproduction in any medium, provided the original work is properly cited.
License URL: https://creativecommons.org/licenses/by/3.0/

==============================
Behavioural Neurology 23 (2010) 253–254
DOI 10.3233/BEN-2010-0308
IOS Press

### Motion influences emotion, but also structural facial features recognition

A. Sedda[a,*], V. Manfredi[a], A. Parente[a] and G. Bottini[a,b]
[a]*Psychology Department, University of Pavia, piazza Botta 6, 27100 Pavia, Italy*
[b]*Cognitive Neuropsychology Center, Niguarda Ca' Granda Hospital, Milan, Italy*

### 1. Introduction

Although motion is crucial for ecological facial perception, Facial Emotion Recognition (FER) studies mainly used static pictures as stimuli. Recent experiments highlight that motion could improve FER by providing dynamic information, facilitating configural processing and enhancing the perception of change [2]. However, motion may have a generic effect for facial features recognition, rather than a selective facilitation for FER, especially according to new models of face perception [4]. For instance, simple internal characteristics movements, such as eyes looking to the right, may take advantage of a dynamic presentation as well as facial emotion expressions [5]. Previous studies mainly used static [2] or degraded [7] pictures for control tasks, not directly comparing FER to simple physical features recognition. The aim of our work is to explore the role of motion in both emotional and structural facial features recognition.

### 2. Methods

To test the hypothesis of a wide role of motion we administered participants with two classical tasks used in FER (emotional static, emotional dynamic) and two ad hoc developed control tasks (Facial Structure Recognition – FSR, static and dynamic). Pictures for all tasks were taken from the black and white Ekman and Friesen series [3], to guarantee repeatability. Emotional categories were disgust, anger, fear, happiness and sadness. For the FSR task, different individuals have been selected from the Ekman and Friesen series and images were modified, using Gimp 2.6.4 (Windows version, Copyright © 1995–2008), to obtain 5 non-emotional categories: left or right mouth changes (raising of one side), left or right eyes changes (gaze direction), and raised eyebrows. We modified stimuli for the dynamic conditions using the morphing technique [6]. Abrosoft FantaMorph (V. 3.7.1) was used to create the dynamic stimuli from two static images, the neutral face and the 100% expression, both emotional and non-emotional. Each dynamic sequence comprised 30 frames/s and the global duration of the video presented to participants was 1.50 seconds. Static pictures were displayed for the same duration as the video sequence.

Forty-two normal participants volunteered the experiment. In all conditions, participants were instructed to press a button as soon as they recognized the image displayed (reaction time recording) while saying the correct answer (accuracy recording). These parameters were taken as dependent variables.

### 3. Results

First, we performed a 2 (Task: FER versus FSR) by 2 (Motion: Static Versus Dynamic) repeated measure ANOVA, collapsing separate categories together to highlight the effect of task.

### *3.1. Accuracy*

We found a main effect of Task ($p < 0.001$) and Movement ($p < 0.001$) and a significant interaction

*Corresponding author. E-mail: sedda.anna@gmail.com.

ISSN 0953-4180/10/$27.50 © 2010 – IOS Press and the authors. All rights reserved between factors ($p < 0.001$). Post hoc comparisons for the FSR task showed greater accuracy in the static than in the dynamic condition ($p < 0.001$). Moreover, FER accuracy was greater in both the static ($p < 0.001$) and dynamic condition ($p = 0.001$) compared to the FSR task.

### 3.2. Reaction time

Task showed a significant main effect ($p < 0.001$). Furthermore, a significant interaction between Task and Movement ($p < 0.001$) was found. Post hoc comparisons showed faster responses in the FER static respect to the dynamic condition ($p = 0.001$), while in the FSR faster responses in the dynamic condition ($p < 0.001$). Moreover, FER showed slower responses than FSR task in the dynamic condition ($p < 0.001$).

A second analysis, involving one task at time, further explores the effect of Motion on Category of the FER (disgust, fear, anger, happiness and sadness) and FSR (eyes left and right, mouth left and right and eyebrows) tasks.

### 3.3. Accuracy

Main effects of Movement were found only in the FSR ($p < 0.001$) and not in the FER task. Both tasks showed a significant interaction between Category and Movement ($p < 0.001$). Particularly, we found a significant difference in fear ($p = 0.001$) and disgust ($p = 0.01$) recognition between the static and dynamic condition, although in opposite directions: better accuracy for dynamic fear and static disgust. Moreover, we found an improvement in accuracy recognition in the dynamic presentation for mouth changes both on the left ($p < 0.001$) and on the right ($p < 0.001$).

### 3.4. Reaction time

While both tasks showed a main effect of Movement ($p < 0.001$), a significant interaction between Movement and Category was found only in the FSR task ($p < 0.001$). For the FER task the most striking result is the significantly slower reaction times in the dynamic condition for all the emotional categories ($p = 0.001$), which is opposite to the results of the FSR task, in which dynamic presentation leads to faster answers ($p = 0.001$).

### 4. Discussion

Our results highlight an effect of motion, affecting facial features recognition, widespread across structural changes and relevant also for non-emotional features. The novelty of our study is the presence of a control task involving non-emotional facial changes, developed from the widely used Ekman and Friesen series. The results from this direct comparison are still preliminary and have some limitations, and further research is needed. Nonetheless, our results are in agreement with the most recent model of face perception [4], in which it is proposed a *dual route* of face processing with two distinguished but intercommunicating streams, respectively decoding invariant and changeable aspects of faces. Although in FER studies on brain damaged patients [1] it can be useful to evaluate only emotions *per se*, we speculate that in normal subjects it would be better to consider all the non-rigid facial movements that primary involve the internal features of faces (eg. nose, mouth, eyes) [5], such as speech production movements and eye gaze direction, as social signs permeate most kinds of facial motions [5].

### References

[1] R. Adolphs, D. Tranel and A.R. Damasio, Dissociable neural systems for recognizing emotions, *Brain and Cognition* **52**(1) (2003), 61–69.

[2] Z. Ambadar, J.W. Schooler and J.F. Cohn, Deciphering the enigmatic face: the importance of facial dynamics in interpreting subtle facial expressions, *Psychological Science* **16**(5) (2005), 403–410.

[3] P. Ekman and W. Friesen, Pictures of facial affect. Palo Alto, CA: Consulting Psychologists Press, 1976.

[4] J.V. Haxby, E.A. Hoffman and M.I. Gobbini, The distributed human neural system for face perception, *Trends in Cognitive Sciences* **4** (2000), 223–233.

[5] D.A. Roark, S.E Barrett, M.J. Spence, H. Abdi and A.J. O'Toole, Psychological and neural perspectives on the role of motion in face recognition, *Behavioral and Cognitive Neuroscience Reviews* **2**(1) (2003), 15–46.

[6] W. Sato and S. Yoshikawa, The dynamic aspects of emotional facial expressions, *Cognition and Emotion* **18** (2004), 701–707.

[7] W. Sato and S. Yoshikawa, Enhanced Experience of Emotional Arousal in Response to Dynamic Facial Expressions, *Journal of Nonverbal Behavior* **31** (2007), 119–135.
